# Supplementary material for: Structural basis for tuning activity and membrane specificity of bacterial cytolysins
Source: Nat Commun. 2020 Nov 16;11:5818. doi: 10.1038/s41467-020-19482-6 (PMC7669874; doi:10.1038/s41467-020-19482-6)
Supplement: Supplementary file 1 — Supplementary Information [file 41467_2020_19482_MOESM1_ESM.pdf]

Structural basis for tuning activity and

membrane specificity of bacterial cytolysins

Authors: Nita R. Shah[1], Tomas B. Voisin[1], Edward S. Parsons[2], Courtney M. Boyd[1], Bart W. Hoogenboom[2,3], Doryen Bubeck[1]*

Affiliations:

[1] Department of Life Sciences, Sir Ernst Chain Building, Imperial College London, London, SW7 2AZ, UK

[2] London Centre for Nanotechnology, University College London, London, WC1H 0AH, UK

[3] Department of Physics and Astronomy, University College London, Gower Street, London, WC1E 6BT, UK

*__Contact Information:__ d.bubeck@imperial.ac.uk

ONLINE SUPPLEMENTARY INFORMATION

SUPPLEMENTARY TABLES

Supplementary Table 1. Primer pairs used to generate CDC mutations. Mutated nucleotides are underlined and in bold.

Mutation	Primer pairs (5' to 3')
**ILY**	
A221C	GCTGAAATCGGCGCCAAATTTGC**A**TTGCAGCTGGGACATACTTTG<br>CAAAGTATGTCCCAGCTGCAA**T**GCAAATTTGGCGCCGATTTCAGC
N404C	TTGGTTTCAATATAGTCCGTATTG**C**ACTGGATGGTTGCAATGCTATTATC<br>GATAATAGCATTGCAACCATCCAGT**G**CAATACGGACTATATTGAAACCAA
Y208C	CCGGCTCGCATGCAGT**G**CGAAAGCATCTCTGC<br>GCAGAGATGCTTTCG**C**ACTGCATGCGAGCCGG
D322C	GCAGGCGGCCATC**TG**TGCCGTCGTGAAAGGCGC<br>GCGCCTTTCACGACGGCA**CA**GATGGCCGCCTGC
L340C	GGAATACGAAAACATC**TGC**AAAAACACCAAAATCACG<br>CGTGATTTTGGTGTTTTT**GCA**GATGTTTTCGTATTCC
N342C	CGAAAACATCCTGAAA**TG**CACCAAAATCACGGC<br>GCCGTGATTTTGGTG**CA**TTTCAGGATGTTTTCG
E337A,<br>N338A,<br>K341A,<br>N342A	GGTACGGAATACG**C**A**GC**CATCCTGG**GC**A**GC**CACCAAAATCACGG<br>CCGTGATTTTGGTG**GC**T**GC**CAGGATG**GC**T**G**CGTATTCCGTACC
Y336K,<br>L340K	GCTGGTACGGAA**A**A**G**GAAAACATC**AA**GAAAAACACCAAAATCACGG<br>CCGTGATTTTGGTGTTTTTC**TT**GATGTTTTC**C**T**T**TTCCGTACCAGC
Y336W	GAAAGCTGGTACGGAAT**GG**GAAAACATCCTG<br>CAGGATGTTTTC**CC**ATTCCGTACCAGCTTTC
N338W	GGTACGGAATACGAA**TGG**ATCCTGAAAAACACC<br>GGTGTTTTTCAGGAT**CCA**TTCGTATTCCGTACC
L340W	CGGAATACGAAAACATC**TG**GAAAAACACCAAAATC<br>GATTTTGGTGTTTTTC**CA**GATGTTTTCGTATTCCG
N342W	CGAAAACATCCTGAAA**TGG**ACCAAAATCACGGC<br>GCCGTGATTTTGGT**CCA**TTTCAGGATGTTTTCG
**PLY**	
W278K,<br>L282K	CCACAGACCGAG**GC**GAAGCAGATT**GC**GGACAATACGGAAG<br>CTTCCGTATTGTCC**GC**AATCTGCTTC**GC**CTCGGTCTGTGG

K279A, Q280A, D283A, N284A	CAGACCGAGTGG**GC**G**GC**GATTTTG**GC**C**GC**TACGGAAGTGAAGG<br>CCTTCACTTCCGTA**GC**G**GC**CAAAATC**GC**C**GC**CCACTCGGTCTG

Supplementary Table 2. All CDC variants used in this study

Variant	Mutations	Disulfide bonds	Source
**ILY**			
WT$^{\text{charge+}}$	Y336K, L340K		This study
WT$^{\text{no charge}}$	E337A, N338A, K341A, N342A		This study
WT$^{\text{Y336W}}$	Y336W		This study
WT$^{\text{N338W}}$	N338W		This study
WT$^{\text{L340W}}$	L340W		This study
WT$^{\text{N342W}}$	N342W		This study
prepore$^{\#}$	I104C, G244C	C104-C244	Boyd *et al*.[1]
prepore$^{\text{L340C}}$	I104C, G244C, L340C	C104-C244	This study
prepore$^{\text{N342C}}$	I104C, G244C, N342C	C104-C244	This study
prepore$^{\text{charge+}}$	I104C, G244C, Y336K, L340K	C104-C244	This study
HB1lock	A221C, N404C	C221-C404	This study
HB1lock$^{\text{charge+}}$	A221C, N404C, Y336K, L340K	C221-C404	This study
HB1lock$^{\text{no charge}}$	A221C, N404C, E337A, N338A, K341A, N342A	C221-C404	This study
HB1lock$^{\text{Y336W}}$	A221C, N404C, Y336W	C221-C404	This study
HB1lock$^{\text{N338W}}$	A221C, N404C, N338W	C221-C404	This study
HB1lock$^{\text{L340W}}$	A221C, N404C, L340W	C221-C404	This study
HB1lock$^{\text{N342W}}$	A221C, N404C, N342W	C221-C404	This study
HB2lock	Y208C, D322C	C208-C322	This study
HB2lock$^{\text{charge+}}$	Y208C, D322C, Y336K, L340K	C208-C322	This study

HB2lock$^{no\ charge}$	Y208C, D322C, E337A, N338A, K341A, N342A	C208-C322	This study
HB2lock^{Y336W}	Y208C, D322C, Y336W	C208-C322	This study
HB2lock^{N338W}	Y208C, D322C, N338W	C208-C322	This study
HB2lock^{L340W}	Y208C, D322C, L340W	C208-C322	This study
HB2lock^{N342W}	Y208C, D322C, N342W	C208-C322	This study
**PLY**			
WT$^{charge+}$	W278K, L282K		This study
WT$^{no\ charge}$	K279A, Q280A, D283A, N284A		This study

#prepore refers to the ILY early prepore solved by cryoEM in this study

Supplementary Table 3. Collection specifications of cryoEM data sets

	Data set 1	Data set 2	Data set 3
Microscope	Titan Krios	Titan Krios	Titan Krios
keV	300	300	300
Camera	Falcon III	Falcon III	Falcon III
Collection mode	Linear	Linear	Linear
Pixel size (Å)	1.4	1.4	1.4
Stage tilt during collection	0°	0°	30°
Number of frames	39	39	39
Integration time (s)	1.00	1.00	1.22
Total dose ($e^-/Å^2$)	66.8	69.4	60.1
Defocus range (µm)	-1.9 to -3.1	-1.9 to -3.1	-1.9 to -3.1
Number of micrographs	5202	5959	6878

Supplementary Table 4. Data processing, refinement, and validation statistics for early prepore ILY-CD59 model

	ILY early prepore (EMDB- 11172) (PDB 6ZD0)
**Data processing**	
Symmetry imposed	C1*
Initial particle images (no.)	105,448
Final particle images (no.)	51,041
Map resolution (Å)	4.6
FSC threshold	0.143
Map resolution range (Å)	4.0-7.0
**Refinement**	
Initial model used (PDB code)	4BIK
Model resolution (Å)	5.2
FSC threshold	0.5
Model resolution range (Å)	4.0-6.0
Map sharpening *B* factor ($Å^2$)	-220
Model composition	
Non-hydrogen atoms	7974
Protein residues	1611
B factors ($Å^2$)	
Protein	346
R.m.s. deviations	
Bond lengths (Å)	0.005
Bond angles (°)	1.176
**Validation**	
MolProbity score	2.08
Clashscore	9.95
Poor rotamers (%)	0.00
Ramachandran plot	
Favored (%)	89.73
Allowed (%)	10.14
Disallowed (%)	0.13

* See details of local symmetry in Methods

SUPPLEMENTARY FIGURES

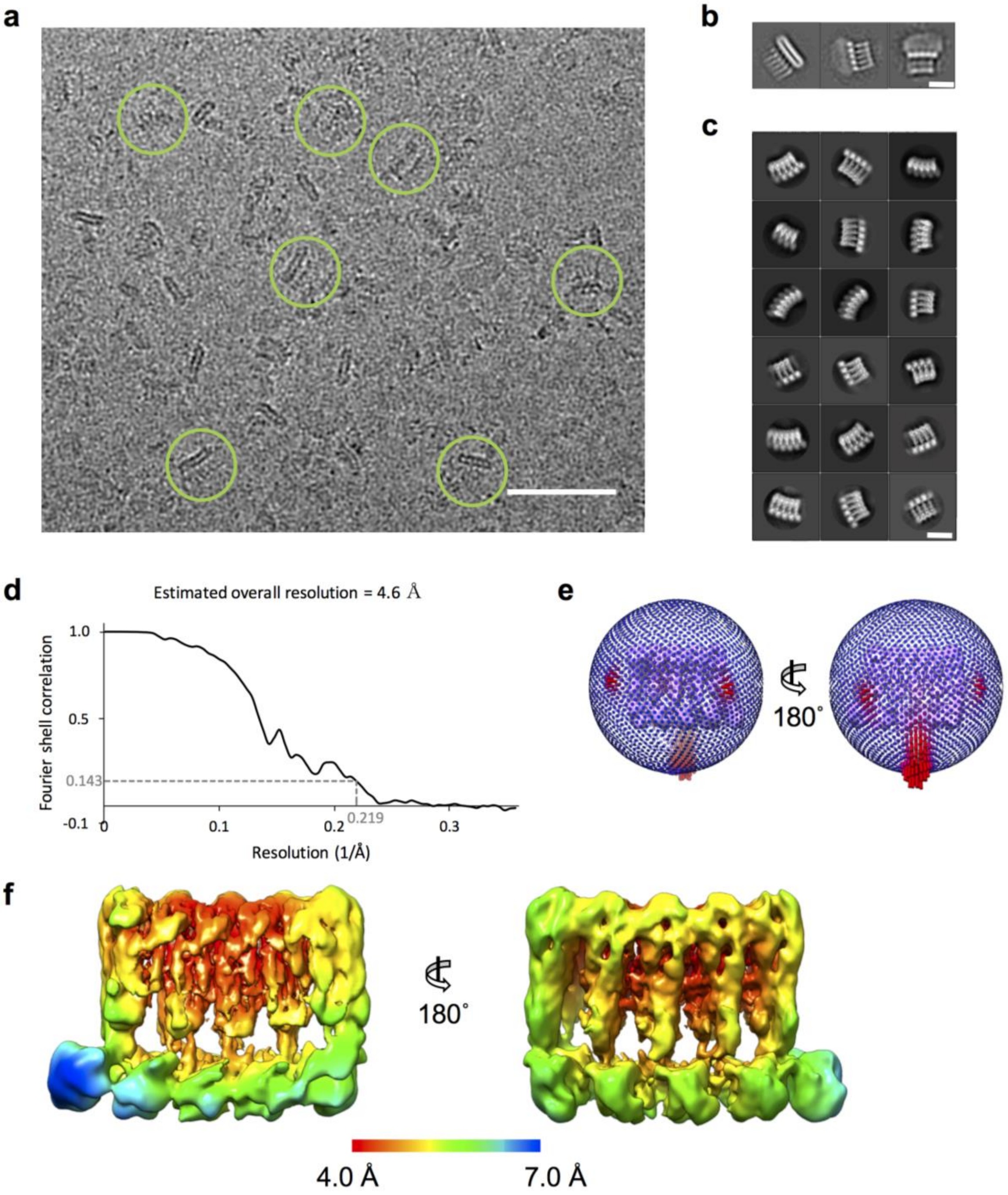

Supplementary Figure 1. CryoEM reconstruction of ILY early prepore in complex with CD59. (a) Representative particles from an electron micrograph (comprised of a total set of 18,039 micrographs) of early prepore ILY on graphene oxide-coated holey carbon grids. Several particles circled in green. Scale bar, 500 Å. (b) Selected early 2D class averages that include nanodiscs, before particles were re-centered and masked to exclude nanodisc signal. Scale bar, 100 Å. (c) Selected 2D class averages from final classification. Scale bar, 100 Å. (d) Mask-corrected Fourier shell correlation (FSC) curve computed from unfiltered half-maps in RELION. (e) Angular distribution for the reconstruction. Height of the cylinder at each projection direction is proportional to the number of particle images, ranging from blue (fewer images) to red (more images). (f) Local resolution filtered map colored according to resolution, ranging from 4.0 to 7.0 Å. Data shown in (d-f) correspond to the final reconstruction obtained following the bottom right branch in Supplementary Figure 2 (purple).

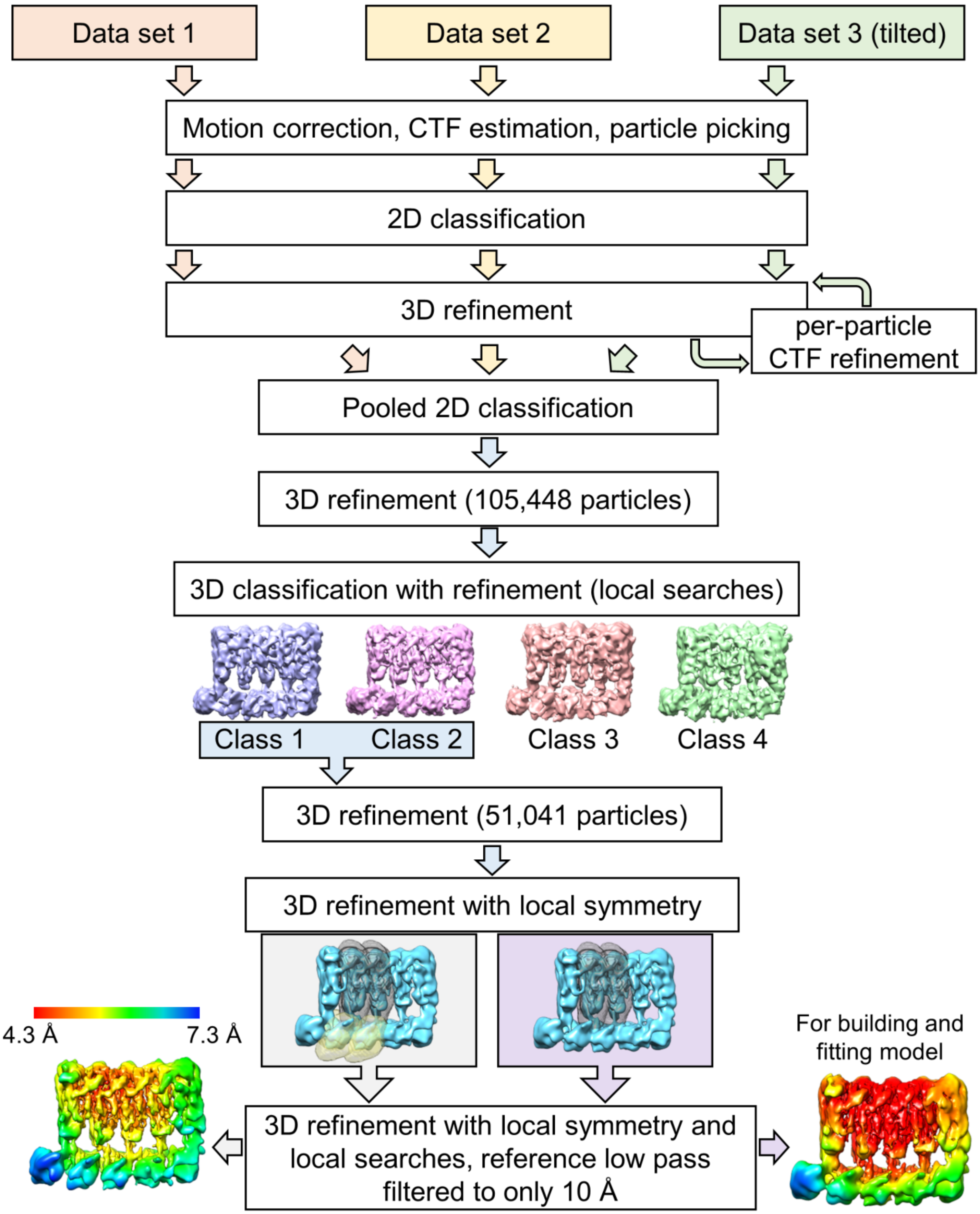

Supplementary Figure 2. Data analysis and reconstruction strategy for early prepore ILY oligomer. Particles were picked from three data sets (red, yellow, and green) whose micrograph movies were individually preprocessed (motion correction and CTF estimation). 2D classification

of extracted particles from each data set removed poor particles, and the remaining particle sets were refined using an initial model generated in RELION. For Data set 3, per-particle CTF-refinement was performed to account for variation in z-height across the tilted micrographs, followed by another 3D refinement. All particles were then pooled (105,448 particles) and subjected to refinement to generate a consensus map. 3D classification with refinement separated these particles into 4 classes. Particles from Class 1 and 2 were combined (51,041 particles) and refined using local symmetry operators. In the right branch (purple background), the top regions of two monomers (gray) were assigned one set of symmetry operators. In the left branch, the bottom region of two monomers (yellow) were assigned an additional set of symmetry operators. A second round of 3D refinement with these respective symmetry operators was performed using local searches and a reference low pass filtered to 10 Å. The local resolution-filtered density reconstructions (bottom left and right) are shown colored according to the same resolution scale.

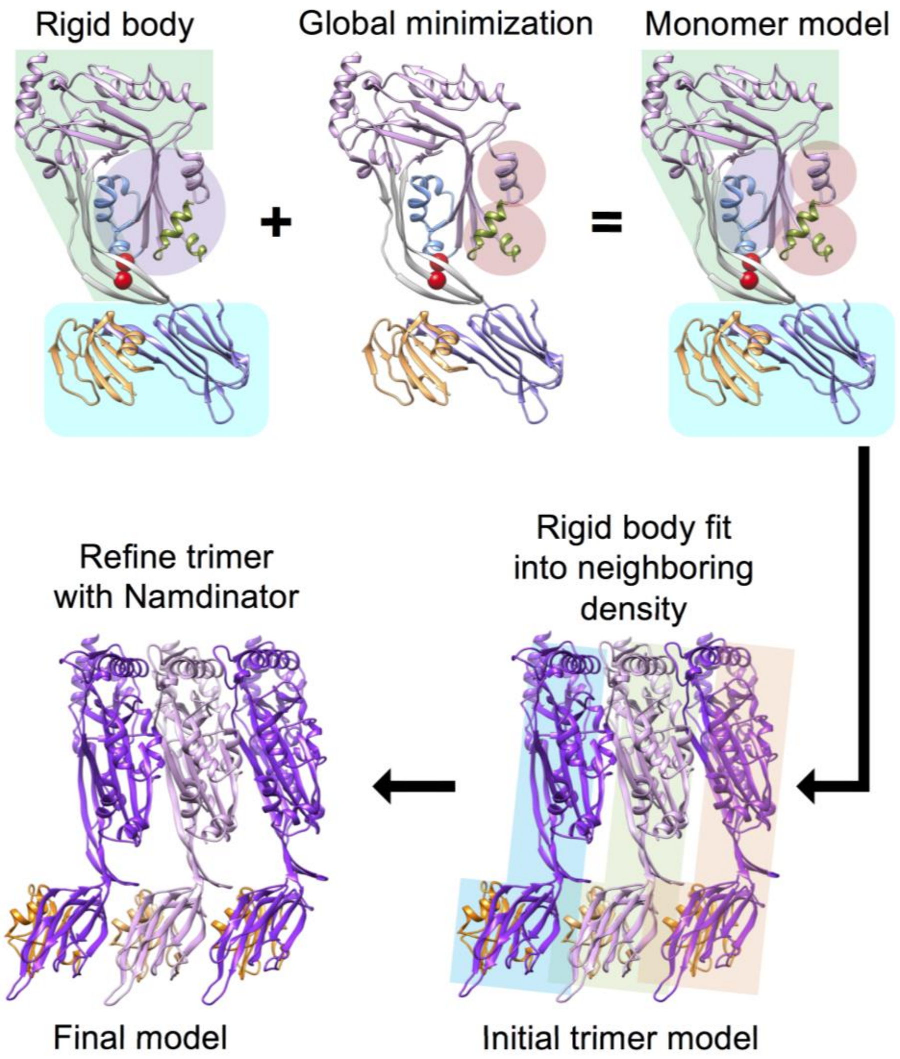

Supplementary Figure 3. Schematic summary of model building and refinement of the ILY prepore oligomer. The structure of soluble, monomeric ILY bound to CD59 (PDBID 4BIK[2]) was first rigid body fit into the central monomer of the pentameric cryoEM reconstructed density, rigid bodes are highlighted in green, blue and purple (top left). The HB2 helices and newly built helix-turn-helix motif (highlighted in red, top middle) were further refined with global minimization and secondary structure restraints, and added to the rigid body-fit model to generate the monomer model (top right). The monomer model was rigid body fit into the neighboring densities to generate the initial trimer model (bottom right). Residues within the oligomer were further refined using Namdinator selecting the implicit solvent option, which combines Phenix

real_space_refine with molecular dynamics simulations. Sidechains were then removed followed by per-residue B-factor refinement to generate the final model (PDBID 6ZD0). The Phenix cryoEM validation tool[3] was used to assess the validation statistics of the final model (Supplementary Table 4) and to generate average and per residue cross-correlation coefficients comparing the map and model (Supplementary Fig. 4). All structures depicted in this schematic are of the final PDBID 6ZD0 model.

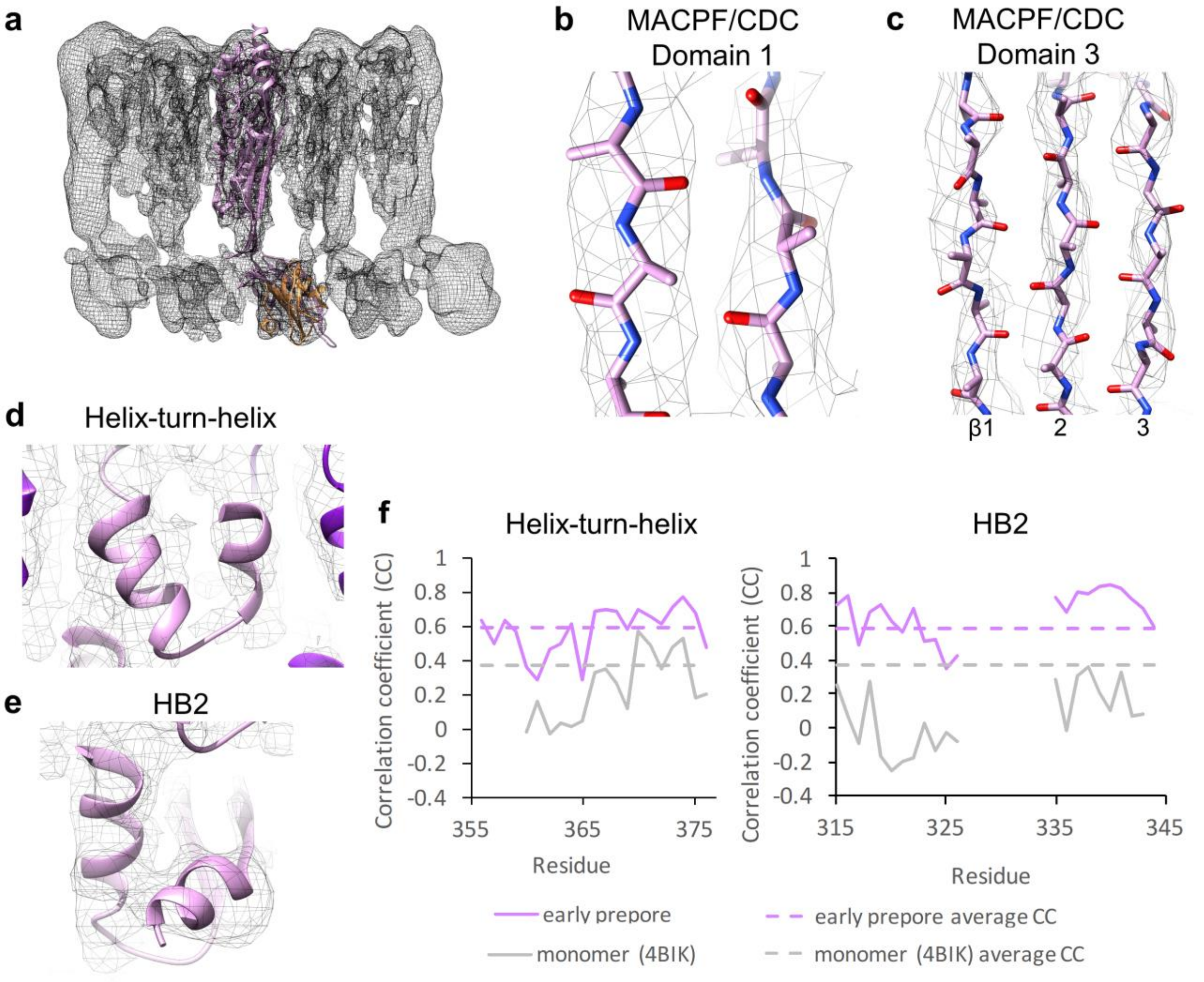

Supplementary Figure 4. Structural models of ILY (pink) and CD59 (orange) overlaid with the local resolution filtered cryoEM map (gray mesh). (a) Overlay of the ILY-CD59 early prepore model fit in the central monomer. (b) Quality of the map showing separation of β-strands within MACPF/CDC domain 1 (residues 258-261, 297-300). (c) Map and model overlay showing β-

strands of MACPF/CDC domain 3 (residues 205-211, 249-256, and 301-308). (d) Close-up of the ILY helix-turn-helix motif (residues 356 -373) showing agreement of the map and model. (e) Close-up showing the quality of the fit for vertical and horizontal helices (h-helix) of HB2 (residues 315 to 342). (f) Average map to model correlation coefficient of the ILY early prepore model with our cryoEM reconstruction was calculated using the tool phenix.validation_cryoem. A per-residue correlation coefficient for the helix-turn-helix motif and HB2 helices are highlighted. The average correlation coefficient for the template reference structure used to initiate model building and refinement (PDBID 4BIK[2]) is shown for comparison.

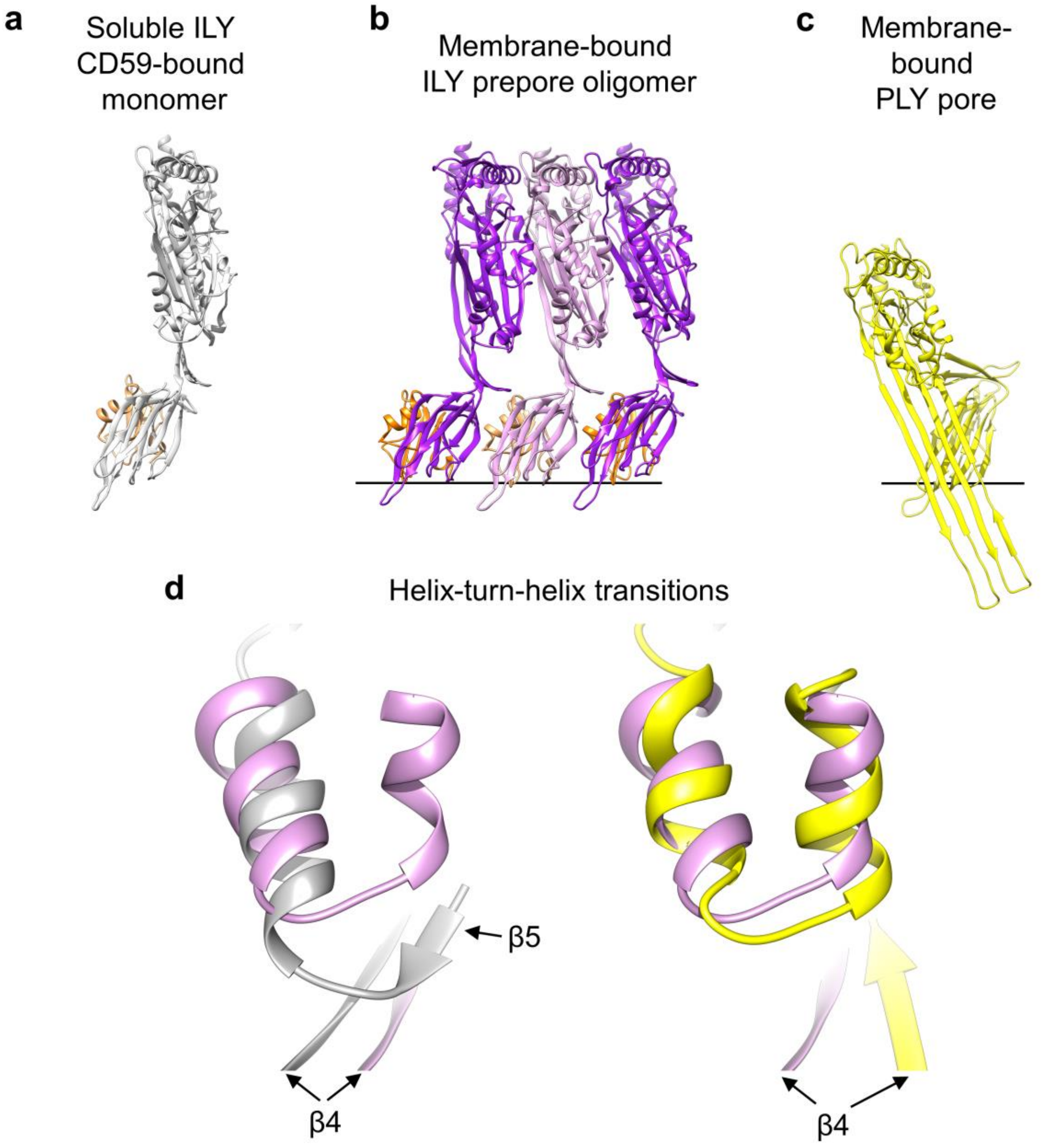

Supplementary Figure 5. Comparison of (a) soluble, monomeric ILY bound to CD59 (gray, PDBID 4BIK[2]), (b) membrane-bound ILY prepore oligomer (pink and purple, PDBID 6ZD0) and (c) membrane-bound PLY pore (yellow, PDBID 5LY6[4]). (d) Left panel displays the transition of ILY β-strand 5 (soluble ILY monomer, gray) into the helix-turn-helix motif present in the ILY early prepore (pink). Right panel overlays the ILY early prepore (pink) and PLY pore (yellow) helix-turn-helix motifs. Consequently, as the CDC transitions from soluble monomer to membrane-inserted pore, β-strand 4 of the MACPF domain swings backwards.

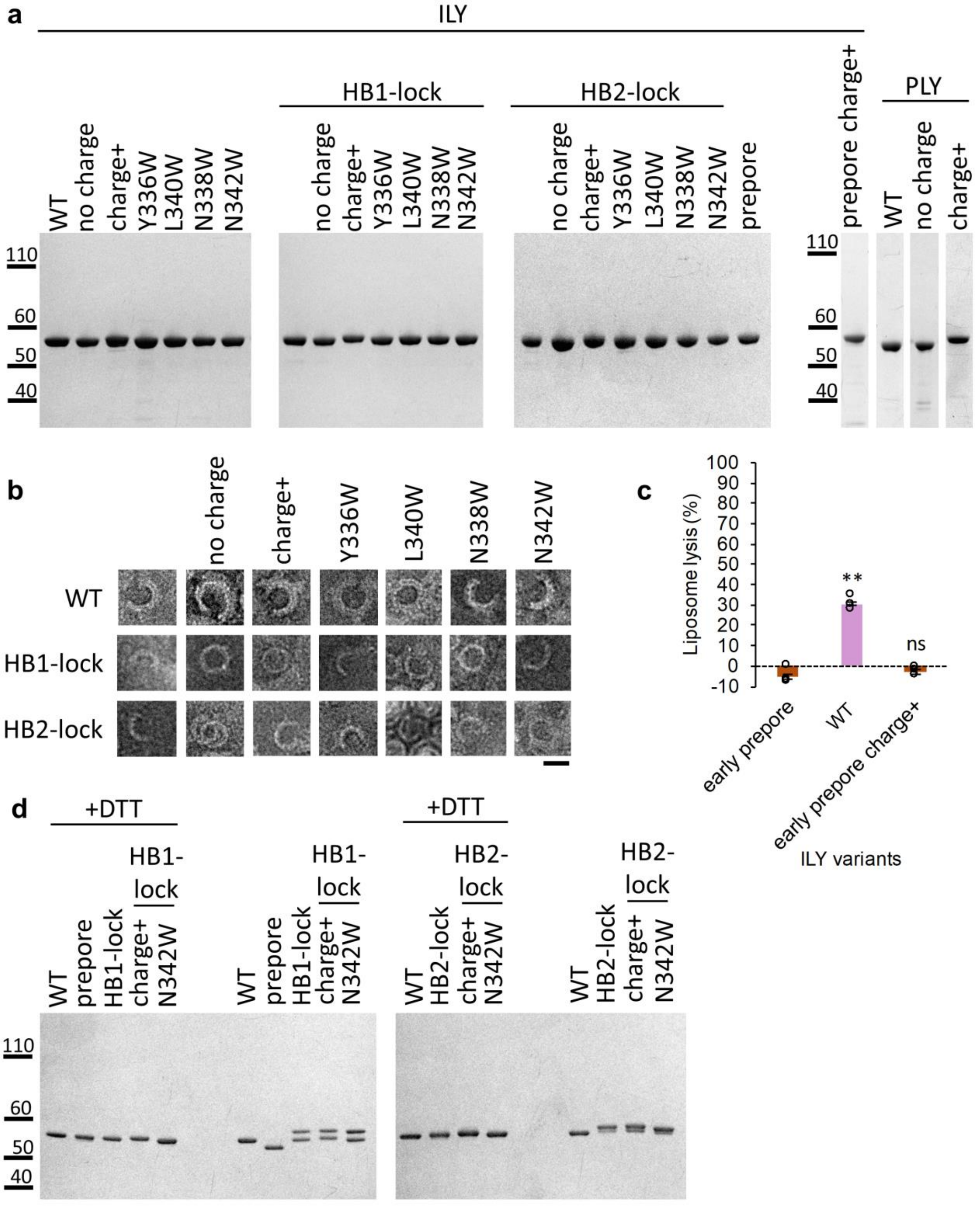

Supplementary Figure 6. Assessing quality of the ILY and PLY variants. (a) Protein purity was assessed by SDS PAGE analysis. The three gels on the left were compositionally similar and run as a group, therefore the numbering on the left represents size in kDa for these three gels.

The four gels on the right are compositionally similar and run as a group, therefore the numbering in the middle represents size in kDa for these four gels. (b) Correct protein folding was assessed by imaging arc or ring formation on DOPC:cholesterol (1:1 molar ratio) monolayers in the presence of CD59 by negative stain EM. Each arc or ring image is representative of data seen in three different micrographs. Scale bar (bottom right), 300 Å (c) Calcein-based liposome lysis assay. Wild type ILY (WT) and ILY early prepore variants were tested for their ability to lyse cholesterol containing liposomes decorated with CD59. Early prepore charge+ refers to the early prepore variant with h-helix mutations: Y336K, L340K. All experimental readings were normalized to a control reading of liposomes in buffer. Statistical significance displayed above each bar is for comparison with early prepore. Individual data points from three independent experimental assays are shown as circles. Error bars represent standard deviation, with the center of the bar indicating the mean value. P-value significance determined by one-way ANOVA with a Bonferroni post-test: ns, not significant; **, $p < 0.01$. (d) Disulfide bond formation was assessed by gel shifts in SDS PAGE under reducing (+DTT) and non-reducing conditions. Under non-reducing conditions, a fraction of HB1-lock and HB2-lock variants remain unshifted. These two gels were compositionally similar and run as a group, therefore the numbering on the left represents size in kDa for both gels. This is a representative gel of two independent reduction experiments.

REFERENCES

1. Boyd, C.M. et al. Disentangling the roles of cholesterol and CD59 in intermedilysin pore formation. *Sci. Rep.* **6**, 38446 (2016).
2. Johnson, S., Brooks, Nicholas J., Smith, Richard A.G., Lea, Susan M. & Bubeck, D. Structural basis for recognition of the pore-forming toxin intermedilysin by human complement receptor CD59. *Cell Rep.* **3**, 1369-1377 (2013).
3. Afonine, P.V. et al. New tools for the analysis and validation of cryo-EM maps and atomic models. *Acta Crystallogr. D* **74**, 814-840 (2018).
4. van Pee, K. et al. CryoEM structures of membrane pore and prepore complex reveal cytolytic mechanism of Pneumolysin. *eLife* **6**, e23644 (2017).
