# Supplementary material for: Structural basis for tuning activity and membrane specificity of bacterial cytolysins
Source: Nat Commun. 2020 Nov 16;11:5818. doi: 10.1038/s41467-020-19482-6 (PMC7669874; doi:10.1038/s41467-020-19482-6)
Supplement: Supplementary file 2 — Reporting Summary [file 41467_2020_19482_MOESM2_ESM.pdf]

nature research

Corresponding author(s): Doryen Bubeck

Last updated by author(s): October 9, 2020

Reporting Summary

Nature Research wishes to improve the reproducibility of the work that we publish. This form provides structure for consistency and transparency in reporting. For further information on Nature Research policies, see our Editorial Policies and the Editorial Policy Checklist.

Statistics

For all statistical analyses, confirm that the following items are present in the figure legend, table legend, main text, or Methods section.

n/a	Confirmed	
☐	☒	The exact sample size (*n*) for each experimental group/condition, given as a discrete number and unit of measurement
☒	☐	A statement on whether measurements were taken from distinct samples or whether the same sample was measured repeatedly
☐	☒	The statistical test(s) used AND whether they are one- or two-sided<br>*Only common tests should be described solely by name; describe more complex techniques in the Methods section.*
☐	☒	A description of all covariates tested
☐	☒	A description of any assumptions or corrections, such as tests of normality and adjustment for multiple comparisons
☐	☒	A full description of the statistical parameters including central tendency (e.g. means) or other basic estimates (e.g. regression coefficient) AND variation (e.g. standard deviation) or associated estimates of uncertainty (e.g. confidence intervals)
☐	☒	For null hypothesis testing, the test statistic (e.g. *F*, *t*, *r*) with confidence intervals, effect sizes, degrees of freedom and *P* value noted<br>*Give P values as exact values whenever suitable.*
☒	☐	For Bayesian analysis, information on the choice of priors and Markov chain Monte Carlo settings
☒	☐	For hierarchical and complex designs, identification of the appropriate level for tests and full reporting of outcomes
☒	☐	Estimates of effect sizes (e.g. Cohen's *d*, Pearson's *r*), indicating how they were calculated

Our web collection on statistics for biologists contains articles on many of the points above.

Software and code

Policy information about availability of computer code

Data collection	CryoEM were collected with EPU software version 1.5.1.50 and 1.12.0.79 (Thermofisher Scientific).
Data analysis	CryoEM data was analyzed with published softwares RELION versions 3.0 and 3.1, CTFFIND version 4.1.13, MotionCor2, Cryolo version 1.5.4, Phenix version 1.18, Namdinator, Chimera version 1.13, and Coot version 0.9. AFM data was analysed with Gwyddion version 2.55. All other data was analyzed with Microsoft Excel version 15.24.

For manuscripts utilizing custom algorithms or software that are central to the research but not yet described in published literature, software must be made available to editors and reviewers. We strongly encourage code deposition in a community repository (e.g. GitHub). See the Nature Research guidelines for submitting code & software for further information.

Data

Policy information about availability of data

All manuscripts must include a data availability statement. This statement should provide the following information, where applicable:

- Accession codes, unique identifiers, or web links for publicly available datasets
- A list of figures that have associated raw data
- A description of any restrictions on data availability

CryoEM data and corresponding structural model that support the findings of this study have been deposited in EMDB (EMD-11172) and PDB (6ZD0) databases, respectively. We used previously published structural data deposited in the EMBD (EMD-4118) and PDB (4BIK, 1S3R, 5CR6, 5LY6, 5AOE, 1M3I, 4CDB, 3HVN, 5IMY, 6CB8, 5NMN, 1IJV) databases in our analysis. The raw data that supports the non-structural findings of this study are available from the corresponding author upon reasonable request. All ILY and PLY mutants created in this study are made available at Addgene.

Field-specific reporting

Please select the one below that is the best fit for your research. If you are not sure, read the appropriate sections before making your selection.

☒ Life sciences ☐ Behavioural & social sciences ☐ Ecological, evolutionary & environmental sciences

For a reference copy of the document with all sections, see nature.com/documents/nr-reporting-summary-flat.pdf

Life sciences study design

All studies must disclose on these points even when the disclosure is negative.

Sample size	N=3 is standard practice for assessing statistical significance. For experiments where more variability was observed a sample size of 6 was used to assess statistical significance.
Data exclusions	For structural studies, particles were excluded based on 2D and 3D classification. Classes that were consistent with a 5-mer oligomer were taken forward for further analysis. No data was excluded from any other experiments.
Replication	Data was replicated over two independent experiments. All attempts at replication were successful.
Randomization	Not relevant to this study, since samples were not allocated into experimental groups.
Blinding	Not relevant to this study, since there were no group allocations in this study.

Reporting for specific materials, systems and methods

We require information from authors about some types of materials, experimental systems and methods used in many studies. Here, indicate whether each material, system or method listed is relevant to your study. If you are not sure if a list item applies to your research, read the appropriate section before selecting a response.

Materials & experimental systems

n/a	Involved in the study	
☒	☐	Antibodies
☒	☐	Eukaryotic cell lines
☒	☐	Palaeontology and archaeology
☒	☐	Animals and other organisms
☒	☐	Human research participants
☒	☐	Clinical data
☒	☐	Dual use research of concern

Methods

n/a	Involved in the study	
☒	☐	ChIP-seq
☒	☐	Flow cytometry
☒	☐	MRI-based neuroimaging
